# Towards a fiercely urgent expansion of laboratory medicine in Africa

**DOI:** 10.4102/ajlm.v10i1.1785

**Published:** 2021-12-17

**Authors:** Iruka N. Okeke

**Affiliations:** 1 Department of Pharmaceutical Microbiology, Faculty of Pharmacy, University of Ibadan, Ibadan, Nigeria

The *African Journal of Laboratory Medicine* (AJLM) has published its 10th annual volume! Our journal was established in 2011 on the tails of rapid and unprecedented growth of laboratory medicine in Africa that began in the current century.^1^ The journal’s first nine volumes published articles covering virtually all scientific, social science and practice specialties in, or studying, laboratory medicine. The AJLM has also proudly curated six forward-looking special issues focused on the Strengthening Laboratory Management Towards Accreditation programme (2014),^2^ quality assurance in point-of-care testing for HIV (2016),^3^ the role of the laboratory in global health security (2016),^4^ strengthening tuberculosis diagnostic networks in Africa (2017),^5^ African laboratories in antimicrobial resistance surveillance (2018)^6^ and the future of diagnostics (2020).^7^

It has been especially rewarding to steer our journal in the last four years of its first decade and to simultaneously watch the reputational quality my predecessors established maintained, while growing what is now the pre-eminent laboratory medicine journal on the African continent. Working during a pandemic has made the last two extraordinarily productive years doubly strenuous, for us and all other biomedical science editorial offices. At the end of 2020, our valiant, but inaccurate, assessment was that our essential pandemic contributions might be temporary.^8^ One year later, it is clear that the battle against coronavirus disease 2019 (COVID-19) will continue for some time to come and, therefore, how we operate now must become part of our future.

It is perhaps fitting that I reflect on the fact that, while the scope of our journal is very broad, 8 of 32 articles published in Volume 10, excluding this editorial, relate to the pandemic. COVID-19 articles make roughly a quarter of our pages; this reflects the enormous effort that laboratory medicine researchers and practitioners have put into the response. It serves as one of many records of the heavy burden the pandemic has placed on the African continent, which has made giant strides in developing laboratory medicine in the last decade. There are 24 non-COVID-19 articles in Volume 10 (2021), while the number of such articles published pre-pandemic in 2018 and 2019 was 17 and 29. Thus, we have added pandemic scientific publishing onto our usual, and growing, AJLM output. Nonetheless, we have not diverted all our focus towards the response to a single disease. For centuries, Africa has been fighting infections without necessary laboratory medicine support;^9^ a new journal was born to document the African laboratory’s role a decade ago,^1^ but this substantial upsurge in published papers – just one reading of diagnostic progress – happened now. Therefore, in a sense, at least some of our growth was pandemic-driven.

The pandemic also forces us to accept that there is some predictability that new diseases of unpredictable nature could emerge in the future, and that scientific knowledge and laboratory capacities must be extended to meet them. A more recent, but equally pressing, rise in non-communicable disease prevalence adds to the essentiality of, and demand for, effective laboratory medicine and improved health systems, which can offer needed laboratory support for high-quality care. For all of these reasons, we should expect our journal to grow geometrically in the next decade. Our editors, readers and authors must keep pace with the rising magnitude and increased diversity of laboratory operations that support clinical care.

No doubt, growth is essential, but how quickly does it need to take place? Synthetic evidence has shown that most patients in Africa still do not have access to needed diagnostics for managing their illnesses,^10^ despite advances in laboratory medicine on the continent in the past two decades. Even for severe acute respiratory syndrome coronavirus 2 (SARS-CoV-2), where we have seen phenomenal growth and a truly impressive scale of testing and laboratory-led surveillance,^11,12^ access to laboratory medicine for COVID-19 on the African continent is still suboptimal for public health and patient management. The experts that author and read our articles are working frenetically to close the gap. The answer to the question of ‘How quickly?’ must be ‘Very’.

The response needed to close Africa’s diagnostic gaps is not only critically important, it is fiercely urgent. The value of health systems, and the will to maintain them, will become recognisable only when the best evidence, often from laboratories, forms the basis for patient treatment and management.^13^ Moreso, every missed or mistreated illness due to diagnostic and laboratory insufficiency is a prolonged or, often, fatal illness.

Once the need for any change becomes apparent, harder pushes are needed to make change happen and sometimes events coincide to produce a landscape that will help ensure that the effort invested will field results. The pandemic, although a time of great difficulty, has created such a time for laboratory medicine. We must leverage current impetus for better laboratory diagnostics to avert future disaster. One of the oft-quoted speeches of the United States’ Martin Luther King Jr enjoins us to push when opportunity stands in front of emergency:

We are now faced with the fact that tomorrow is today. We are confronted with the fierce urgency of now. In this unfolding conundrum of life and history, there ‘is’ such a thing as being too late. This is no time for apathy or complacency. This is a time for vigorous and positive action.^14^

As our journal, and the African Society for Laboratory Medicine that owns it, move into their second decades, we must consider the need to grow not only expansively, but also quickly and purposefully as part of a multipronged, continent-wide and locally proclaimed effort to enhance health for Africans.^15^ We must just as fiercely move from the motivating rhetoric of another place and era to *now*, and we must hold our selves, as well as others, to account.^16^ This is particularly true because the current pandemic has shown us that rapid but rigorous growth is possible in laboratory medicine and powerful tools exist to help get us there.^17,18^ We have now seen that the price of ‘rapid’ growth is insignificant compared to the cost of not growing responsively is far responsively. We must, therefore, plan to scale up laboratory medicine accordingly.
